# Cases of Peritonitis; with Remarks

**Published:** 1830-09

**Authors:** Hugh L. Hodge

**Affiliations:** one of the Physicians of the Almshouse Infirmary.


### PERITONITIS.

*Cases of Peritonitis; with Remarks.* By HUGH L. HODGE, M.D. one of the Physicians of the Almshouse Infirmary.*

MORBID anatomy has greatly illustrated pathology: it has several times brought to light extensive consequences of a disease, whose existence even had not been previously suspected, and has often demonstrated the frequency of a complaint which had been thought very rare. Peritonitis has been detected in many cases by the knife of the anatomist, where its occurrence had escaped the suspicions of the mere practitioner, and is now therefore regarded as a more frequent complaint than had hitherto been supposed. Many individuals have no doubt perished from this and other inflammatory affections, whose death has been attributed to debility.

These errors arise from an imperfect diagnosis. There are no symptoms peculiar to either acute or chronic inflammation of the peritoneum, and, as the physician must form his opinion from the group of symptoms presented, so he is liable to be deceived even in the acute forms of the affection. Thus, Dr. CHAPMAN has informed me that two of our most observant and experienced physicians for a long time attended a lad for peritoneal inflammation, whose body after death presented no evidences of such an affection; the pleura and lungs having been the seat of irritation and disease. I have met with several severe cases of apparent peritonitis, with swelling, tension, and pain of the abdomen, decubitus on the back, dread of the least pressure, even from the bedclothes, which proved to be a rheumatic affection of the integuments. On the contrary,

* North American Med. and Surg. Journal.

when peritonitis has actually existed, the symptoms have sometimes been so obscure or complicated as to cause it to be mistaken for colic, diarrhœa, dysentery, enteritis, &c. In chronic peritonitis the signs are still more deceptive. I have been called in consultation by a respectable physician to a case of this character, which had been for months treated as ascites; and in this and many other cases dissection has revealed the most extensive inflammation of the peritoneum, with sero-purulent effusions, and universal adhesion of opposing surfaces, where no suspicion of the disease had existed during life. The reverse I have also found to be true: that the practitioner who, during life, has suspected, from the peculiar combination of symptoms, a chronic inflammation of the abdominal cavity, has in vain searched for the consequences of such inflammation after death. "A very obscure variety of peritonitis is one which is only indicated by a tumefaction of the abdomen, joined to habitual constipation and to sensibility without fluctuation. A more obscure degree is that in which there is swelling, with only occasional constipation; and, finally, peritonitis has been announced merely by a simple tumefaction *(ballonement)* of the belly."* It is needless to observe how wholly insufficient are such symptoms to identify the nature or seat of an abdominal disease.

There is still another source of deception respecting the diagnosis of peritonitis, arising from its not unfrequent complication with inflammation of the mucous membrane of the bowels, particularly of the large intestines. At our infirmary I have met with several cases in which the acute form of peritonitis has suddenly supervened on chronic inflammation and ulceration of the intestines. Such cases are exceedingly distressing, from the agony to which the patient has been usually subjected, and, proving speedily fatal, have afforded the opportunity by dissection for understanding the true condition of the tissues involved. In other instances the phlogosis of the serous membrane has been the primary disease, while the affection of the mucous tissue has accidentally supervened. But, in chronic affections of these membranes, whether resulting from a common cause or as consequents the one of the other, I should say, from the observations I have made, that the symptoms of enteritic disease are alone prominent; those of the peritoneal affection seldom attract attention, while the former is comparatively a manageable disease, and the latter, in

* Dict. abrégé des Sciences Médicales. Art. Peritonite.

its confirmed state, is almost incurable. "Peritonitis, whether acute or chronic, particularly the latter, is of all complaints the most fatal."* In treating, therefore abdominal inflammations, it becomes necessary to ascertain, if possible, all the tissues which are involved; for it may happen, as will be presently exemplified, that the inflammation of one tissue may be subdued, while that of another may destroy the patient. If the diagnosis be difficult, and the disease usually fatal, greater attention to minutiæ is required, that, by an early detection, the proper time for the successful employment of our powerful therapeutical agents may not be irremediably lost. Inflammation should be considered as a curable disease, and however difficult its management may be rendered from the circumstances of the case, the scientific physician should not despair in his attempts to discover its insidious approaches or to arrest its progress.

In confirmation of some of these remarks, I shall adduce two cases very analogous to each other; one of which I have the pleasure of presenting in detail, the account having been drawn up by Dr. Nott, house physician to the infirmary during my superintendence, from notes taken at the bedside of our patient. One or both cases would appear to confirm some important facts in the history of peritonitis, and to afford matter for reflection.

1. That peritoneal inflammation may exist unsuspected: there is reason to believe that, in both the following cases, this inflammation had preceded the mucous affection, which last first induced the patients to apply for relief.

2. That severe and dangerous inflammation in the mucous and serous tissues of the intestines may coexist.

3. That enteritic tympanitis is intimately associated with, if not a necessary attendant on, peritonitis: it forms a very important diagnostic mark.

4. That suppuration may occur on a serous surface without ulceration; the pus having a "laudable" character.

5. That the pus may in these serous tissues be circumscribed by adhesions, and the abscess thus formed may, as in ordinary cases, advance by progressive absorption to the surface of the body, or open into an adjoining intestine.

6. That ulceration may commence originally in a serous tissue; a point doubted by some pathologists.

7. That the peritoneal inflammation may continue its ravages, while the mucous inflammation may be arrested, or

* Op. cit. p. 468.

even subdued; and this without any very positive indications.

8. That the sympathies are so little excited in chronic peritonitis, that the inflammation may pass through its different stages, so that gangrene may result before the patient is destroyed.

9. That, in a therapeutical point of view, the local application of cold promises to be useful as a palliative, if not as a permanent remedy. No measure was effectual in relieving the pain in Graham's case until cold was resorted to.

Case I. was that of a mulatto man, about thirty years of age, of a delicate constitution. He attributed his illness to exposure to wet and cold; and some days after the commencement of his complaint was brought to the infirmary. He then complained of the usual symptoms of dysentery. His abdomen being, however, enlarged (tympanitic) and painful, especially on pressure, the patient was treated by means of cathartics, anodynes, and demulcents: the symptoms greatly ameliorated, and he appeared convalescent. In this situation, contrary to advice, he left the infirmary, and returned to his usual mode of living. He, however, was in a few days readmitted into the institution, and again treated as suffering from dysentery. He complained of severe griping pains, irritation of the rectum, tenesmus, with frequent inclination to stool. The discharges per rectum were, however, peculiar; presenting the appearance of yeast in colour and consistence, as if a large quantity of sero-mucous fluid had been evacuated, without any admixture of bile or feces. The abdomen was tympanitic, and pain was excited by pressure. By the use of alteratives (calomel, ipecacuanha, and opium in combination,) and laxatives, assisted by cold affusions to the abdomen and blisters to the lower extremities, the symptoms of mucous irritation were again subdued. The stools became bilious and feculent, and there seemed to be no reason why the patient should not recover. He remained, however, weak and miserable, was much emaciated, without appetite; had a slight paroxysm of fever in the afternoon; the abdomen was tympanitic, but not painful, except when much pressure was made. In this state he continued, with little apparent alteration, for several days: his strength, however, gradually diminished, and he died.

On dissection, there were evidently some vestiges of mucous inflammation; but bilious feces were found in the intestines, and there was nothing in this tissue sufficient to account for the death of the patient. The peritoneal surface, however, was universally inflamed; lymphatic depositions connected all the viscera of the abdomen; while pure pus was found in circumscribed cavities among the convolutions of the intestines and the folds of the mesentery. In the latter situation a large abscess was formed. Several portions of the peritoneum were of a dark or livid colour, and easily torn, as if in a gangrenous condition.

Case II. *as detailed by my friend,* Dr. Nott. Edward Graham, an Irish labourer, ætat. thirty, of intemperate habits, was admitted on the 19th of June, 1828, with mania a potu threatening. Pulse at that time was a good deal excited, tongue a little furred, slight headach. Was ordered a dose of calomel and jalap, which he said purged him freely: after this he was sitting up, and was thought to be convalescent, until the evening of the 21st, when he was seized with sharp pains in the abdomen, and a frequent disposition to stool, without being able to evacuate any thing. Abdomen was a good deal swollen. These symptoms were attributed to some impropriety in diet, and, thinking the case nothing more than a common colic, without further examination Calomel gr. x., Pulv. Opii gr. i. were prescribed.

22d. He was a good deal better, but still complained of his abdomen, which, on examination, was found to be very tender and considerably swollen; pulse frequent, full, and soft; tongue more foul; skin rather warm and dry; bowels not opened by the last medicine.—Ordered cups to abdomen. Calomel gr. ii., Pulv. Opii gr. ss. M.; also common enema.

23d, eight o'clock a.m. No improvement; skin hot; face flushed; pulse frequent, open, with slight tension; tongue of a dirty cream colour, brownish and dry in centre, edges florid; abdomen still swollen and tender.—Ordered a scruple of Calomel, to be followed by Oil, till bowels are freely opened; sixty leeches to abdomen, and warm fomentations.

Three o'clock p.m. No relief: had two stools; pain in abdomen acute, (compared it to knives running through him;) pulse more tense; skin very hot.—Ordered venesection; bore only eight ounces, which was cupped and buffy. Enema and fomentations repeated.

Ten p.m. Better: has been perspiring freely; bowels well opened; stools copious, soft, feculent, and offensive; pulse 104 and full; abdomen slightly relieved. Ordered sixty leeches and an enema of senna and salts. Fomentations continued.

24th, nine A.M. The injection given last night brought away four or five copious stools; feels considerably relieved this morning; pulse and skin nearly natural; tongue much the same. Diet gruel.

Three P.M. Not so well: pulse more frequent and tense; abdomen again quite tender. Ordered sixty leeches and fomentations.

Ten P.M. Considerably relieved: had several stools during the day.

25th, nine A.M. Improving: pulse pretty full; has a little tenderness of abdomen; tongue still foul. Calomel gr. x., Ext. Coloc. comp. gr. x. M.

Ten P.M. Medicine did not operate. Was ordered, during the afternoon, an injection, which opened his bowels several times. Tongue the same; pulse irritated.

26th. Appears to be a good deal better: tongue cleaner; has little tenderness remaining. Thinks himself well, and, contrary to advice, persists in leaving the house: he took his discharge and went out.

30th. Admitted again, after being out four days: most probably has been eating and drinking imprudently, although he denies it; has acute tenderness on right side of umbilicus; abdomen swollen, particularly at the part where the tenderness is seated;* pulse eighty-eight, small, and tense; tongue clean and florid at the edges; skin natural; has a cough; has had, since his discharge, frequent slimy stools, with griping. Ordered eighty leeches to pained spot.

July 1st. No better: abdomen very tender; tongue covered with a cream-coloured fur; skin cool; tormina and tenesmus, but no stools; pulse quick and tense. Calomel gr. x. to be followed by oil.

2d. Bowels were freely opened yesterday; pulse frequent, quick, and tense; skin not hot, but has a husky feel; appearance of tongue and tenderness of abdomen much the same. Ordered forty leeches and fomentations.

* I would remark, that the general cavity of the abdomen was but slightly swelled in this case, but that an ovoid tumor formed below and to the right of the umbilicus, presenting to the eye the appearance of an abscess formed among the muscles, but, on examination by percussion and with the fingers, the swelling evidently depended on gas in the bowels. This tumor was the seat of the patient's severe sufferings; and, in my clinical remarks, I mentioned to the pupils in attendance that I considered it a case of peritonitis, in which adhesions had occurred, in all probability causing the accumulation of air, and that there was reason to suspect an abscess would form.

Evening. Not so well as in the morning: pulse 100, small, quick, and tense; skin hot; abdomen very tender. Ordered affusions of cold water on abdomen, and injections of cold water; heat of skin and pulse was reduced by the cold, and he felt better after it.

3d. Much the same this morning; had several watery stools during the night. Cold water continued. Ordered blisters to ankles, and Ipecac. gr. $\frac{1}{4}$ q. b. h.

4th. The blisters yesterday produced so much excitement that it was found necessary to remove them before they had fully drawn. Feels better this morning; pulse ninety-nine and quick; tongue the same; abdomen not near so painful to the touch, but is still protuberant; skin good; stools more consistent and natural.—Cold and ipecacuanha continued.

6th. Improving: little or no tenderness of abdomen remaining.—Treatment continued.

7th. The swelling of the abdomen is becoming conical in form, pointing near the umbilicus; has a tympanitic sound on percussion, and, when kneaded, the gurgling of air is heard in the intestine; pulse irritable.—Ordered a blister to the tumefied portion of the abdomen.

9th. Still complains of frequent inclination to stool: discharges very small and slimy.—Ordered Calomel gr. x.

Patient is becoming feeble: allowed milk and chicken water. Diet heretofore has consisted almost entirely of gruel and gum water.

20th. Has been lingering ever since the 9th without any material change in symptoms. Abdomen until yesterday remained tense and pointed. Dr. Hodge thinks that an abscess has formed in the peritoneum: two blisters have been applied successively, without any marked effect: pulse has continued irritable; febrile exacerbations in the evening; small and frequent evacuations from the bowels. Tonics, astringents, and opiates have been given without any advantage. Yesterday afternoon the tumor of the abdomen subsided: patient says that he had at the time a considerable discharge of fluid and flatus, but, as the stools had been thrown away by the nurse without examination, it could not be ascertained whether or not they contained pus. He is now taking porter and diet to support his strength: all the astringents have failed in checking his bowels; he is also using burnt brandy and galls.

27th. Although the swelling of abdomen subsided on the 19th, a circumscribed hardness remained, extending for several inches around the umbilicus. An opening formed yesterday at the umbilicus, through which during the day about a pint of pure pus was discharged, according to the account of the nurse and patient: the discharge is now trifling, and the patient would be pretty comfortable, if it were not for his diarrhœa and a cough, which increases with the debility. Ordered injections of Sulph. Zinci gr. x. in half a pint of water, to check bowels.

29th. Slept pretty well last night: cough getting worse, and expectoration more difficult, owing to increased debility. Hectic paroxysms followed by copious perspiration: discharge now about two or three ounces daily; had two or three stools during the last twelve hours. Ordered Elix. Vitr. gtt. x. t. in d.; wine whey, and volatile liniment to breast.

30th. Gradually sinking: discharge from umbilicus of a greenish hue, and very offensive: on pressure it comes out, mixed with air and the contents of the intestine. Treatment continued.

August 1st. Died.

*Dissection.* On opening the abdomen, appearances of extensive chronic peritoneal inflammation were seen. The peritoneum every where presented a leaden hue; pretty firm adhesions had formed between the folds of the intestines, between the omentum and intestines, and between the liver and peritoneum lining the diaphragm, &c. The omentum and small intestines, just behind the umbilicus, had become agglutinated, and were united at the distance of two or three inches around the umbilicus to the anterior parietes of the abdomen: a complete sac was thus formed, which contained pus and feculent matter: the abscess extended as far up as the liver, and had but two openings, one at the umbilicus, and another, about the size of a crow quill, into the small gut: the sac was so perfectly formed that no pus was found extravasated amongst the intestines around it. Five or six ulcers, about the size of a ten cent piece, were seen on the *external surface* of the small intestines, but *perforating only the peritoneal coat*, except the one communicating with the abscess: a little abscess was also found near the left groin, in the folds of the mesocolon, around which was poured out about a gill of pus. The mucous coat of the stomach showed some marks of chronic inflammation; that of the small intestines was perfectly healthy, showing the ulcers had commenced on the external or peritoneal surface.

The mucous membrane of the large intestine was extensively diseased; looked as if it had been corroded by strong acid, a great portion of it being removed by ulceration: it was of a light chocolate colour, and much thickened.

N.B. I think it most probable that the abscess opened into the intestine before it did externally: the smallness of the aperture in the intestine will account for the late appearance of fecal matter in the abscess.

